# A Practical Treatise on Mechanical Dentistry

**Published:** 1886-11

**Authors:** 


### Bibliographical.

A Practical Treatise on Mechanical Dentistry.—By Joseph Richardson, M. D., D. D. S. Fourth edition, revised and enlarged. Publishers: P. Blakiston, Son & Co., Philadelphia, 1886. Price, cloth, $4.50; leather, $5.50.

The new edition of this well-known and highly prized work, contains 703 pages and 458 illustrations, some two hundred and sixty pages more than the third edition, and seventy-three more illustrations. One hundred and ninety-three pages are devoted to artificial crowns on roots and bridge-work. Speaking of facial individuality the author remarks: "Each separate feature—as the eye, the nose, the mouth, the teeth, facial contour, complexion, temperament, etc., contribute to this individuality, and no one special feature more, perhaps, than the teeth. There are few more repulsive deformities than those inflicted by the loss of these organs, and none more fatal to the habitual and characteristic expression of the individual. It is the special mission, as it is the first and highest duty, of the dentist to preserve this individuality intact, and an equally imperative duty to restore it as perfectly as possible when impaired. To fulfil in the most perfect manner possible this most difficult of all the requirements of prosthetic practice implies an art culture that is competent to interpret the distinct play of the features associated with individual physiognomies, to differentiate individual temperaments and make available the sculptor's and painter's perceptions of the subtle harmonies of form and color. To the failure or inability to properly comprehend the practical import or significance of individual characteristics, so far as they find expression in the teeth, and the consequent failure to conform our methods of replacement to the imperative requirements of art, may be fairly ascribed the deserved reproach into which prosthetic practice has fallen, and not, as is generally charged, to the employment of any particular material or methods concerned in the mechanical execution of the work." The work now before us is a credit to its author and must prove a valuable assistant to the student as also to the dental practitioner. The publishers should also be commended for the typography and preparation of this volume, which presents a very handsome appearance.

